# Environmental Triggers for Different Behavioral Symptom Clusters

**DOI:** 10.1002/alz.092962

**Published:** 2025-01-03

**Authors:** Carolyn E Pickering, Vicki Winstead, Mustafa Yildiz

**Affiliations:** ^1^ UTHealth Houston, Houston, TX USA

## Abstract

**Background:**

We have previously shown that there are 3 unique behavioral symptom clusters, or groupings of temporally related co‐occurring behavioral and psychological symptoms (BPSD) representing how an individual’s daily BPSD group together relative to their own typical BPSD manifestation across a 21‐day period. To further validate these symptom cluster concepts, we examined whether they are predicted by different environmental triggers.

**Method:**

Family caregivers completed daily diary surveys for 8 consecutive days reporting on the physical, social and care environment in addition to their care recipients different BPSD. Guided by the unmet needs theory, progressively lowered stress threshold theory and environmental theory of BPSD, we used a generalized linear mixed model to assess the effects of different hypothesized environmental triggers on the likelihood of the previously identified symptom clusters manifesting the same day.

**Result:**

The behavioral symptom cluster had significant 2 unique predictors, routine disruption (OR 19, p = 0.005) and skipped care that same day (OR 2, p = 0.005). The psychological‐mood symptom cluster had 5 unique predictors including daily social demands (OR 1.59, p = 0.046), participating in household chores that day (OR 0.38, p = 0.025), number of prescription medications (OR 1.03, p<.001), number of routine over‐the‐counter medications (OR 1.08, p<.001) and larger household composition size (OR 1.28, p<.001). Finally, the psychosis‐like symptom cluster was predicted by use of pain medication that same day (OR 1.51, p = 0.49).

**Conclusion:**

These findings validate that the symptom clusters are mechanistically unique concepts. The findings also highlight modifiable environmental targets that could help to prevent or reduce the occurrence of BPSD. Importantly, findings highlighted the importance of novel concepts like social demands (e.g., decision‐making that is beyond the person with dementia’s capacity) and unmanaged pain in the etiology of BPSD. The increased likelihood of the psychological‐mood cluster due to increased household size may indicate other aspects of the physical environment like noise and available space may impact BPSD.

